# Protecting Health from Climate Change in the WHO European Region

**DOI:** 10.3390/ijerph110606265

**Published:** 2014-06-16

**Authors:** Tanja Wolf, Gerardo Sanchez Martinez, Hae-Kwan Cheong, Eloise Williams, Bettina Menne

**Affiliations:** 1 WHO European Centre for Environment and Health, WHO Regional Office for Europe, Platz der Vereinten Nationen 1, Bonn 53113, Germany; E-Mails: wolft@ecehbonn.euro.who.int (T.W.); sanchezmartinezg@ecehbonn.euro.who.int (G.S.M.); Eloise.Williams@wh.org.au (E.W.); 2 Department of Social and Preventive Medicine, School of Medicine, Sungkyunkwan University, Suwon 440-746, Republic of Korea; E-Mail: hkcheong@skku.edu

**Keywords:** climate change, health, adaptation, WHO European Region, questionnaire survey, Parma Commitments, European Environment and Health Process

## Abstract

“How far are we in the WHO European Region in implementing action to counter the health impacts of climate change?” This was the question posed to representatives of Member States in the WHO European Region of in the WHO working group on health in climate change (HIC). Twenty-two Member States provided answers to a comprehensive 2012 questionnaire that focused on eight thematic areas (governance; vulnerability, impact and adaptation (health) assessments (VIA); adaptation strategies and action plans; climate change mitigation; strengthening health systems; raising awareness and building capacity; greening health services; and sharing best practices). Strong development has been in climate change vulnerability and impact assessments, as well as strengthening health systems and awareness raising. Areas where implementation would benefit from further action are the development of national health adaptation plans, greening health systems, sharing best practices and reducing greenhouse gas (GHG) emissions in other sectors. At the Fifth Ministerial Conference on Environment and Health in Parma, Itatly in 2010, the European Commitment to Act on climate change and health and the European Regional Framework for Action to protect health from climate change were endorsed by the fifty-three European Member States. The results of this questionnaire present the most comprehensive assessment so far of progress made by European Member States to protect public health from climate change since the Parma Conference agreements.

## 1. Introduction

The global climate is changing, affecting human health and well-being. In the near future it will lead to an amplification of current health problems. Climate change will also create new risks and pressures for environmental, social and economic determinants of health. In the World Health Organization (WHO) European Region health effects have already been observed, including more frequent and intense extreme weather events, changes in the geographic range of some infectious disease vectors, longer season and increased concentration of allergenic pollen, and worsened air quality. The WHO Regional Office for Europe supports Member States in protecting health from climate change, by building institutional capacity to assess the health impacts of climate change and by cooperating with national multisectoral committees for identifying and prioritizing adaptation measures.

These activities are in line with World Health Assembly resolution WHA61.19 [1] on climate change and health. They support the implementation of the European Commitment to Act on climate change and health [2] and the European Regional Framework for Action to protect health from climate change [3]. 

Both the Commitment to Act and the European Regional Framework for Action are part of a broad ongoing regional policy effort, the European environment and health process (EHP). The implementation of the EHP is monitored by the European Environment and Health Task Force (EHTF), which has established various working groups to monitor specific aspects of the EHP. One of these groups, the Working Group on Health in Climate Change (HIC), was formed in 2012 and is responsible for monitoring the implementation of the Parma Commitment to Act on climate change and health. HIC originally contained representatives form 31 Member States and five international organizations, whereas by early 2014 Member State representation has grown to 37 Nominated members are from the ministries of either health or environment [2,3].

The European Commitment to Act specifies that Member States will:

- integrate health issues in all climate change mitigation and adaptation measures, policies and strategies at all levels and in all sectors;
- strengthen health, social welfare and environmental systems and services to improve their response to the impacts of climate change in a timely manner, for example to extreme weather events and heat waves. In particular, we will protect the supply of water and the provision of sanitation and safe food through adequate preventive, preparedness and adaptive measures;
- develop and strengthen early warning surveillance and preparedness systems for extreme weather events and disease outbreaks, for example vector-borne diseases, at the animal-human-ecosystem interface, where appropriate;
- develop and implement educational and public awareness programmes on climate change and health, to encourage healthy, energy-efficient behaviours in all settings and provide information on opportunities for mitigation and adaptation interventions, with a particular focus on vulnerable groups and sub regions;
- collaborate to increase the health sector’s contribution to reducing greenhouse gas emissions and strengthen its leadership on energy- and resource-efficient management and stimulate other sectors, such as the food sector, to do the same;
- encourage research and development, for example with tools for forecasting climate impacts on health, identifying health vulnerability and developing appropriate mitigation and adaptation measures [2].

The comprehensive country questionnaire was used as mechanism to monitor the implementation of the climate change part of European Commitment to Act climate and health [2]. The questionnaire developed in alignment with the six strategic objectives of the European Regional Framework for Action [3] had the following objectives:

- assess the current status of Member States regarding activities to mitigate or adapt to climate change;
- identify gaps in the implementation of the Parma commitments;
- provide feedback to European Member States on their action towards protecting health from climate change;
- share experiences and information on best practices in developing and implementing effective adaptation and mitigation measures;
- identify priorities on climate change and health for the next ministerial conference on environment and health.

## 2. Methods: The Questionnaire Survey

The questionnaire covered the following eight areas (see Appendix Table A1) in 45 subquestions:

1. governance
2. vulnerability, impact and adaptation (health) assessments
3. adaptation strategies and action plans
4. climate change mitigation
5. strengthen health systems
6. raise awareness and build capacity
7. green health services
8. sharing best practices.

Prior to sending out the questionnaire, it was tested in one country and the HIC members were asked to provide comments on the questionnaire for validation. The questionnaire was sent via email in English and Russian to 31 nominated HIC members in May 2012. Questionnaires were administered and collected through a focal point in each Member State. Each focal point consulted and shared the questionnaire with the various responsible departments or ministries or with the responsible federal ministries in countries that have federal systems. Russian responses were translated into English and reviewed for their relevance compared to the original questionnaire. We received 22 fully compledted responses with a wealth of additional country-specific information. 

All the answers were coded into numeric form (see Appendix Table A1) and stored into a dataset in Microsoft Excel**^®^**. For this purpose, the WHO Climate, Environment and Health Action Plan and Information System (CEHAPIS) indicator methodology [4] was revised, adapted and consequently used. To describe and analyse the answers, various approaches have were used.

(1) For the quantitative analysis, positive responses to each sub question were scored as one point and considered as progress towards implementing the Parma Commitment. Both negative responses and lack of responses were scored as zero. Whereas the negative responses can be directly correlated with a relative lack of progress towards policy commitments in different areas, the same cannot be said about lack of response for a question. Reasons for not answering individual items can be manifold, and should not necessarily be interpreted as a negative response. Average positive response score by area and presentation of top-scoring countries by items;
(2) Spearman correlation coefficient was used to detect associations between different sections of the questionnaire.

A tentative stratified analysis was carried out based on geographic regions. Responses were analysed by sub region (eastern/western/northern/southern Europe, and central Asia); by European Union (EU) membership status [5] (EU member *vs.* Non-EU member as of January 2012); by Organisation for Economic Co-operation and Development (OECD) membership status [6] (OECD member *vs.* non-OECD member as of January 2012); by presence of a WHO country office (yes *vs.* no as of January 2012); by WHO subregional mortality strata [7]; by United Nations statistical region [8]; and by level of Human Development Index (HDI) [9].

Although a quantitative analysis of the responses is a good way to summarize responses, it cannot show the activities’ explanatory factors behind the specific national situation regarding policy implementation. Looking into the specific responses of a country in a narrative way can provide useful data and rich details, but it is beyond the scope of this article, and thus are saved for a longer upcoming WHO report. Two meetings of HIC have been organized to validate the responses and discuss mode of presentation.

## 3. Results

Of the 31 Member States represented in HIC and that received the questionnaire in 2012, the following 22 countries sent it back completed: Albania, Armenia, Austria, Belarus, Croatia, Denmark, Germany, Hungary, Italy, Kazakhstan, Kyrgyzstan, Lithuania, Montenegro, Norway, San Marino, Serbia, Spain, Slovenia, the former Yugoslav Republic of Macedonia, Turkey, Ukraine, and the United Kingdom. Nine countries did not return it (Belgium, Estonia, France, Israel, Monaco, Netherlands, Russian Federation, Sweden, and Tajikistan).

**Figure 1:**
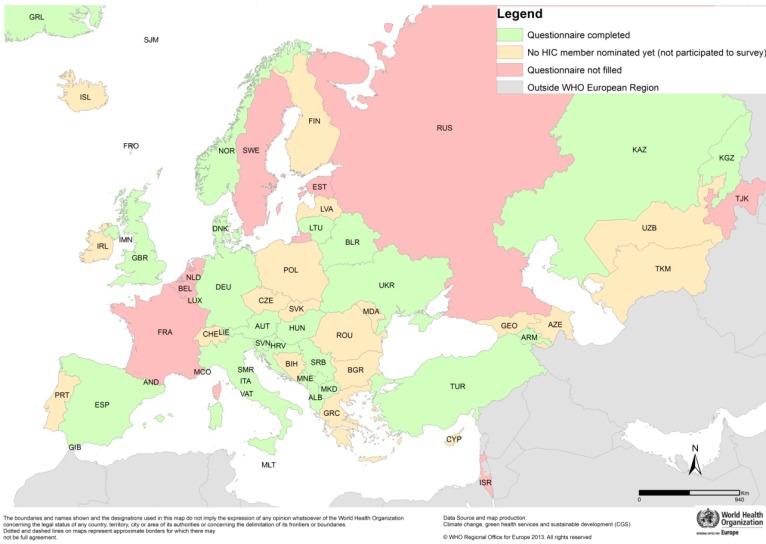
Countries that participated in the questionnaire survey.

The twenty-two countries which had not nominated HIC members by summer 2012 did not receive the questionnaire and were not included in the survey: Andorra, Azerbaijan, Bosnia and Herzegovina, Bulgaria, Cyprus, Czech Republic, Finland, Georgia, Greece, Iceland, Ireland, Latvia, Luxemburg, Malta, Poland, Portugal, Republic of Moldova, Romania, Slovakia, Switzerland, Turkmenistan, and Uzbekistan. The distribution of responders, non-responders and non-participants is displayed in Figure 1.

Of the twenty-two responding countries, eleven are members of the EU and eleven are non-EU countries. The European subregion with the highest representation was southern Europe (nine countries). WHO mortality strata A and B (very low and low child and adult mortality) predominated. The majority of respondents have a WHO country office (14 with country office *vs.* eight without). All surveyed countries except one (plus no data for another) had either high or very high levels of HDI, an indicator that attempts to measure development by combining indicators of life expectancy, educational attainment and income [9]. 

### 3.1. General Evaluation

**Figure 2:**
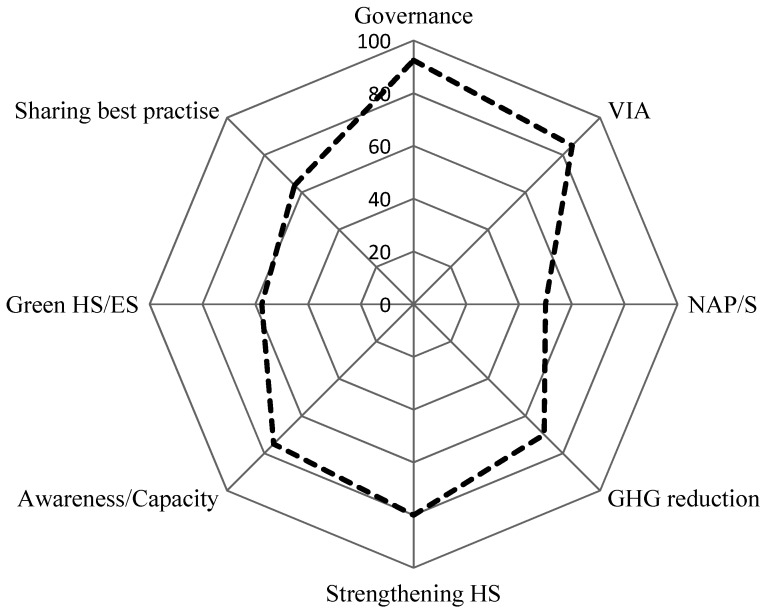
Summary of average percentage response by themes.

Notes: NAP/S: national action plan or strategy; HS/ES: health sectors/environment sectors

Responses of the Member States on the eight topic sections of the questionnaire are summarized in Figure 2, according to the characteristics described below. Questionnaire topics are grouped into conceptually related clusters: governance; vulnerability and impact assessments (VIA) and national adaptation strategies; reducing greenhouse gases (GHG) and greening health services; strengthening health systems and raising awareness; and sharing best practice.

The proportion of countries that responded positively varied by question from as low as 50% to over 90%. The proportion of positive responses and relevant details by question are listed below: 

Governance: In 16 out of 22 (72.7%) countries, the ministry for environment is in charge of climate change. In the remaining countries responsibility for climate change is shared among two or more ministries. In all 22 countries the ministry of health is responsible for climate change and health issues, either independently (16 out of 22 countries) or in conjunction with other ministries . In 21 of 22 (95.5%) countries a multisectoral committee on climate change has been established, whose primary role is to coordinate policies and actions for adaptation and mitigation. Other roles include discussing and verifying reports and adaptation strategies for the United Nations Framework Convention on Climate Change (UNFCCC), developing and implementing policies and action to reduce the exposure to harmful environmental factors, developing technical guidelines as well as recommendations on climate change, monitoring climate change effects, raising awareness and informing other sectors. 

Vulnerability and impact assessments: Member States have made progress in assessing vulnerability and impacts of climate change. 19 of the 22 (86.4%) countries stated that they have conducted national assessments on the impact and vulnerability of climate change. Most efforts are related to the UNFCCC requirement for countries to include such assessments as part of their national communications to the Convention. Seventeen of the surveyed countries (77.3%) have conducted health specific assessments of the impacts, vulnerability, and adaptation to climate change. 

National and subnational adaptation strategies and/or action plans on climate change have been developed in 14 (63.6%) of the countries, and approved by the government in nine. Twelve countries (54.5%) have developed health adaptation plans/strategies on climate change, and in eight countries (36.4%) it has been approved by the government. 

Reducing GHG emissions: with regard to mitigation action and GHG emission reduction, the majority of the Member States responded positively (71.7%). Examples focused in particular on the promotion of energy efficient buildings and promotion of access to safe transport or public transportation. Less progress was reported on shifting to carbon neutral agriculture. Assessment of health benefits of mitigation measures in transport, agriculture, or other sectors has only been reported from seven countries.

Health systems strengthening: on the whole countries responded positively (83.1%—5.7 of 7 items on average). Table 1 shows the examples of measures provided by Member States.

**Table 1:** Measures taken by Member States to strengthen health systems.

Measures Taken by Member States to Strengthen Health Systems	Number of Countries Replying “Yes” or Giving Examples
Strengthened infectious disease surveillance	19
Strengthened of environmental health services (water, sanitation, vaccination)	15
Strengthened health security and implementation of International Health Regulations	15
Strengthened early-warning and disaster response	15
Mainstreamed climate change into public health policy	11
Strengthened primary health care service	13
Ensuring that planning for climate change was included in public health policy	14
Developed integrated climate, environment and health surveillance	7
Built climate-resilient infrastructure	6

Awareness raising: 75% of Member States responded positively. Countries reported a high level of awareness on climate change and a sizeable influence in political developments. The awareness of the relevance of health effects on climate policy was lower.

Green health and environment sectors: Just over half of the Member States responded positively (57.5%). Examples given of activities in this area included energy and carbon management in hospitals, low carbon procurement and food, low carbon travel, transport and access for hospital staff, measuring water and waste saving, indoor energy efficiency measures and renewable energy application. Energy saving in health care facilities was the most common practice in the countries; depending on the state of the sector in each country, this has taken the form of preliminary assessments, infrastructure and retrofitting investments, or incentives for improved performance. 

Sharing best practices: the majority of Member States responded positively (63.8%—5.1 of 8 items on average). Information has mostly been shared on climate change and environmental indicators and pilot projects; regional platforms and websites are commonly used for the sharing of information. 

### 3.2. Regional Variation

WHO European Member States face a high diversity of climate-change-related exposures and vulnerability depending on their geographic location and topography, demographics, economic development and infrastructure. Factors that affect the ability to respond to and prepare for climate-related hazards (including economic development, infrastructure, health systems, and others) are also very diverse across the region. The variability in the questionnaire responses is therefore not surprising. 

However, there were no obvious patterns or differences arising from the stratified analysis. The grouping of countries by United Nations statistical regions as well as HDI and mortality levels did not reveal any consistent pattern and are thus not reported. There were small differences by regional sub grouping but the small sample size precludes any interpretation. OECD countries responded slightly less positively compared to non-OECD countries, especially on VIA activities. Importantly, there were no noticeable differences between EU and non-EU countries regarding their percentage of positive responses. 

In line with the HIC recommendation that good examples (that is, countries that performed at the top in each question) be highlighted in any publications derived from the questionnaire, we feature countries with top scores in different areas are shown in the results below (Table 2). 

**Table 2:** Countries that reached the maximum score by theme.

Country	Governance	VIA	NAP	HS	GHG Redu	GHS	Awareness	Best Practice
Albania	x	x	x					
Armenia	x	x		x	x			
Austria	x	x			x		x	
Belarus	x	x				x	x	x
Croatia	x	x	x	x	x		x	
Denmark		x						
Germany	x	x	x			x	x	x
Hungary	x	x		x				x
Italy	x	x						
Kazakhstan		x						
Kyrgyzstan	x	x						
Lithuania	x	x		x				
Montenegro								
Norway	x	x						
San Marino								
Serbia								
Slovenia						x		x
Spain	x	x	x	x	x	x		x
The former Yugoslav Republic of Macedonia	x	x		x		x	x	x
Turkey	x							
Ukraine	x	x		x				
United Kingdom	x	x					x	
Total	16	17	4	7	4	5	6	6

We also describe examples or characteristics of the activities undertaken in these countries, which contributed to reaching the higher score by theme. 

Some examples of the governance-related activities that one or more of these countries have undertaken include:

- establishing mechanisms for the exchange of information on climate change between agencies;
- allocating resources for implementation at different levels of government (national, regional and local);
- establishing regulatory or legislative instruments to facilitate implementation; and
- involving a wide variety of stakeholders from the inception phase of strategies and plans.

Some examples of VIA activities that one or more of these countries have undertaken include:

- cross-sectoral vulnerability and adaptation evaluations as part of their communications to the UNFCCC;
- economic evaluations of sectoral impacts and adaptation;
- studies evaluating climate effects on vulnerable populations;
- supporting subnational authorities in conducting VIA activities; and
- taking into account emerging threats in their VIA activities.

National action plans (NAPs) of these countries are quite different in scope and focus; some of the NAPs of one or more of these countries include notable features

- The plans have not only general strategies to adapt to climate change, but also health-specific adaptation plans that are approved by the government.
- A capacity building component is included.
- Mitigation in the health sector is considered.
- Inclusion of stakeholders is promoted through participatory processes.

Countries that provided examples of measures to reduce GHG emissions in different sectors (building, transport, agriculture) also stated that they have assessed the health co-benefits of such measures. Examples of strengthening health systems are illustrated in Table 1.

The six countries that reached the maximum score on raising awareness stated that in their country climate change and its health effects are perceived as important in political developments, that public and/or private sector are supportive and capacity on health-related climate change aspects has been built. They also stated that public awareness of climate change and health and of mitigation and adaptation has been raised. Communications regarding extreme weather and climate change and health in general have been developed.

Some examples of activities regarding greening health services in one or more of the high scoring countries included:

- incentives for health care facilities engaging in sustainability activities (e.g. energy efficiency, resource use minimization, *etc*.);
- linking economic, social and environmental sustainability in their long-term strategies for health systems; and
- indicator-based evaluations of sustainability in health systems.

The four countries that reached the maximum score on sharing best practices provided several examples on best practice and have developed projects related to innovation and research. They have also evaluated health damage and adaptation costs. Some interesting actions regarding sharing best practices in one or more of these countries included:

- making information of good practice available publicly on the internet;
- referring information to regional repositories or clearinghouses (e.g., EU); and
- including good practices in comprehensive communications regarding climate change.

### 3.3. Interrelationship between responses

**Figure 3:**
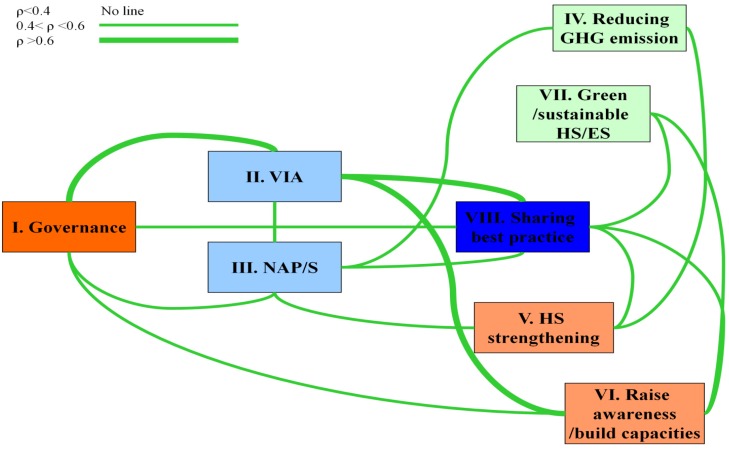
Interrelationship between sections of the national climate change-related activities.

Notes: VIA: vulnerability and impact assessment; NAP/S: national action plan or strategy; GHG: green house gases; HS/ES: health sectors/environment sectors, ρ: Spearman correlation coefficient.

The responses to the questionnaire were further analysed by question to search for interrelationships. A basic statistical analysis of the responses was conducted to explore the relationship of the different elements of climate change protective action. The number of positive responses for each category was summed up and the Spearman correlation coefficient was estimated between categories. Although in this case there is a basic limitation due to the small sample size and potential confounders, it is assumed that the coefficient could represent a rough estimate of the strength of the association between two variables. Figure 3 summarizes the relationship between the categories of the questionnaire. Only statistically significant (*p* < 0.05) relationships with a correlation coefficient equal to or larger than 0.4 are represented by lines, and a thicker line denotes a correlation coefficient higher than 0.6 (Figure 3).

The correlation values suggest a strong relationship between high scores in VIA andgovernance and between VIA and sharing best practice, while the relationship between governance and sharing best practice is less strong. VIA is also related to high scores in raising awareness and building capacity. There are fewer links to reducing GHG emissions and green and sustainable health and environment systems.

## 4. Discussion

This questionnaire was developed to evaluate the implementation of the Parma commitments [2] with regard to climate change. It was quite a complex tool as it required a coordinated effort by multiple agencies in the countries, coordinated by the national focal points. The fact that in 2012 only 31 out of 53 Member States had nominated HIC members, limits the representativeness of the results. Moreover, the response rate of 22 out of 31 underscores that the interest or capacity to retrieve and share information on this topic cannot be taken for granted. More resources and time to follow up on this could lead to more responses from other countries in an eventual second round. 

Accurately measuring the level of implementation of a such complex and far-reaching set of policy commitments is a challenging task. Despite the limitations of this questionnaire, asking relevant stakeholders in Member States is the most practicable way for WHO to assess the level of implementation. Furthermore, the commitment of the countries to this work and their effort to fill in the questionnaire should not be understated. The 22 respondent countries still delivered a wealth of information that was difficult to condense.

Besides the limitations expressed above, a few patterns have emerged: This study provides a regional snapshot on the status of implementation of measures to protect health from climate change in 2012 and 2013. The heterogeneity of Member States’ answers is as substantial as it is unsurprising. The risks of climate change and the sensitivity of health systems and populations vary among and even within, countries. Action or inaction reflects each country’s specific situation, priority setting and decision-making, so any judgment or comparative evaluation is generally avoided in this report. Strong areas of implementation of the Parma commitments on average are governance, the development of vulnerability and impact assessments, strengthening of health systems and raising awareness. The progress in these activities could also reflect the UNFCCC reporting requirements and, to a certain extent, WHO communication, capacity building and training in these areas. Any cut-off point in positive response rates is bound to be arbitrary, but areas where in comparison implementation would seem to benefit from further support are the development of national health adaptation plans, green health services, and sharing best practices.

Some further points have emerged during this assessment.

- Governance mechanisms for climate policy seem well-established, at least in countries with HIC representatives.
- Financial and human resources for climate change health adaptation are mainstreamed into ongoing activities and respective resource planning.
- Although VIA works seems to be an area of relatively strong performance, there are gaps in knowledge and in translating scientific evidence into action.
- The level of governmental approval and uptake of national health adaptation plans is still low.
- Most countries are engaged in mitigation activities of GHG related to buildings, infrastructure and transportation. However, the health co-benefits of these activities were less evaluated.
- Countries reported several activities on health systems strengthening. However, important areas remain lacking, for instance integrated climate, environment and health surveillance, or building climate-resilient health infrastructures.

Several factors add to the difficulties in synthesizing the overall implementation of the Commitment to Act [2], where the diversity of vulnerabilities and national circumstances warrant a country-by-country evaluation. Overall, there are differences that cannot be explained by geographic location, level of development, mortality levels, socioeconomic development or presence of a WHO country office.

Stratified analysis based on geographic regions, EU and OECD membership, WHO mortality strata, presence of WHO country office and HDI category had originally been run. However, few of these attributes were considered useful in describing and explaining heterogeneity in the answers and they have not been reported. On the other hand, the presentation of the answers by clustering the countries according to areas where protection of health from climate change is strong, turned out to be useful for deriving and sharing lessons learnt in the countries.

The overall assessment confirms a finding of the recent 5th assessment report of the Intergovernmental Panel on Climate Change (IPCC): namely that risk management practices have started in a number of European countries, however the type of activities and the complexity and interrelationship between activities is not detailed in the IPCC report. The important role the health sector could play in managing the risks from climate change needs to be emphasized.

## 5. Conclusions

Acknowledging the limitations of this questionnaire in measuring policy implementation, we may derive some general conclusions on the areas covered. The results suggest that Member States are taking action on the Parma Commitment to Act [2] with governance mechanisms for climate policy well-established, at least in countries with HIC representatives. 

Strong areas of implementation of the Parma Commitment to Act [2] include the development of vulnerability and impact assessments, strengthening of health systems and raising awareness. The progress in these activities could also reflect the extended UNFCCC reporting requirements that support WHO communication, capacity building and training in these areas. Vulnerability and impact assessments seem to be a particularly strong area of performance. It is important to note that most VIAs were also performed relatively recently, thus providing a solid foundation for adaptation planning. However, there are still gaps in translating this scientific evidence into action. 

Areas where implementation would potentially benefit from further support are the development of green and environmentally friendly health systems, sharing best practice and reducing GHG emissions in other sectors. There is particular room for improvement regarding governmental adoption of national health adaptation plans. Executive support can dramatically improve the implementation rate of plans, especially when multiple partners are involved.

Further evaluations should include a wider representation of Member States, particularly those who dit not participate in this survey. Incorporation of a wider representation of societal stakeholders in the countries should also be considered. 

## Appendix

**Table A1:** List of questions and quantitative scoring of replies.

Question Number	Topic/Question	Points	Remark
**1**	Governance	4	
**2**	Vulnerability, impact and adaptation assessment	2	
**3**	National and subnational adaptation strategies	4	
**4**	Climate change mitigation	6	
**5**	Strengthen health systems	7	
**6**	Raise awareness—build capacity	10	
**7**	Green health services	4	
**8**	Sharing best practices	8	
		total	**45**
**Question Number**	**Sub Question **	**Points**	**Remark**
**1.1**	Who is in charge of climate change in your country?	1	for answer
**1.2**	Who is in charge of the health aspects of climate change?	1	for answer
**1.3**	Has a multisectoral committee been established to deal with climate change?	1	for yes
**1.4**	Have you identified human and economic resources	1	for yes
		subtotal	**4**
**2.1**	Have you carried out a national assessment of climate change impact, vulnerability and adaptation in your country?	1	for yes
**2.2**	Have you done a national (or regional) health impact, vulnerability and adaptation assessment of climate change in your country?	1	for yes
		subtotal	**2**
**3.1 a**	Have you developed a national adaptation strategy to climate change in your country?	1	for yes
**3.1 b**	Has it been approved by your government	1	for yes
**3.2 a**	Have you developed a national climate change health adaptation strategy or health action plan?	1	for yes
**3.2 b**	Has it been approved by your government	1	for yes
		subtotal	**4**
**4.1**	Do you promote energy efficient buildings?	1	for yes
**4.2**	Do you promote access to safe transport or public transport modes?	1	for yes
**4.3**	Do you promote carbon neutral agriculture practices?	1	for yes
**4.4**	Have you assessed the health benefits of the above measures?	1	for yes
**4.5**	Have mitigation measures in other sectors in your country been taken?	1	for yes
**4.6**	If mitigation measures in other sectors have been taken, have any health effects of those mitigation measures been assessed?	1	for yes
		subtotal	**6**
**5.1**	Have you strengthened public health and health services to cope with climate change? semi-qual.: 10 sub categories	1	for yes
**5.2**	Have you enhanced disease surveillance and early warning of climate sensitive diseases? semi-qual.: sub categories	1	for yes
**5.3**	Have you developed early warning systems for extreme weather events and have you developed appropriate health sector response plans in the areas below? semi qual.: 12 sub categories	1	for yes
**5.4**	Have you strengthened health sector engagement in emergency planning for extreme weather events and have you developed cross-sector plans? semi qual.: 12 sub categories	1	for yes
**5.5**	Have you improved monitoring of climate sensitive environmental determinants of health	1	for yes
**5.6**	Have you developed a cross-sector approach on climate change adaptation?	1	for yes
**5.7**	Do you intend to address health benefits/damages (e.g., by conducting health impact assessment)	1	for yes
		subtotal	**7**
**6.1**	Is climate change perceived as important in political developments in your country?	1	for yes
**6.2**	Are health effects of climate change of high relevance in political processes?	1	for yes
**6.3 a**	Is the level of support for policies targeting climate change and related effects on health high in public and private sector of the society? Public sector	1	for public
**6.3 b**	Is the level of support for policies targeting climate change and related effects on health high in public and private sector of the society? Private sector	1	for private
**6.4**	Do you have enough information at your disposal on climate change and its impact on health with regard to your country?	1	completed answer
**Question Number**	**Special Question**	**Points**	**Remark**
**6.5**	Have you built capacity and developed a workforce on climate change and health-related aspects	1	for yes
**6.6**	Have you raised public awareness about climate change and health and mitigation and adaptation measures?	1	for yes
**6.7**	Have you developed communication messages for extreme weather events to be released with an early warning for such an event?	1	for yes
**6.8**	Have you developed communication plans for key messages on climate change and health for other sectors and the general public?	1	for yes
**6.9**	What are the main messages on protecting health from climate change you would like to communicate?	1	for example
		subtotal	**10**
**7.1**	Greening health services	1	for example
**7.2 a**	Can you list a few examples of measures that have been taken	1	for legislation
**7.2 b**	Have local measures in any health care facilities been taken, like training and organizing the workforce?	1	for measures
**7.2 c**	Have local measures in any health care facilities been taken, like training and organizing the workforce?	1	for evaluation
		subtotal	**4**
**8.1**	Can you share information on best practice with regard to: 6 choices	1	for yes
**8.2**	Have you developed projects or aspects related to innovation and research?	1	for yes
**8.3**	Evaluation of health damage and adaption costs: Have you estimated the costs of climate change and/or the health damage costs	1	for yes
**8.4**	Do you measure and evaluate trends in climate change, environment and health indicators	1	for yes
**8.5**	What do you measure? And to whom do you report?	1	for any measurements
**8.6**	Are you aware of pilot projects in your country on climate change and health? semi qual.: examples of pilot projects	1	for example
**8.7**	Which of the results would you promote to share with other European Member States?	1	for any results
**8.8**	Do you make your information available on the EU adaptation clearinghouse?	1	
		subtotal	**8**
		**total**	**45**
